# Disseminated Intravascular Coagulopathy following Induced Second Trimester Curettage Abortion

**DOI:** 10.5402/2011/591760

**Published:** 2010-10-20

**Authors:** Paulami Guha

**Affiliations:** ^1^Department of OBGYN, St. Bernard Hospital and Health Care Center, 326 West 64th Street, Chicago, IL 60621, USA; ^2^Department of OBGYN, Roseland Community Hospital, 45 W 111th Street, Chicago, IL 60628, USA

## Abstract

*Background*. This is a case of 18-year-old adolescent girl admitted with profuse vaginal bleeding following induced second trimester curettage abortion at 13 weeks of gestation. *Case*. Her transvaginal sonogram detected retained products of conceptus, and her blood reports revealed a full blown picture of DIC. Dilatation and evacuation was done after initial resuscitation with packed RBCs and platelet concentrates. She had an uneventful recovery period. *Conclusion*. DIC is an extremely life-threatening condition which can occur as a very infrequent complication of second trimester abortion.

## 1. Introduction

This is a case of 18-year-old African American female presenting with profuse per vaginal bleeding three days following an induced second trimester curettage abortion. She was diagnosed to be a case of disseminated intravascular coagulation disorder due to retained products of conception.

## 2. Case Presentation

This 18-year-old (gravida 2 parity 1 living issue 1) African American female was brought by the paramedics in the emergency department with history of profuse bleeding per vagina since morning. She had an induced abortion done by dilatation and curettage 3 days ago at 13 weeks of gestation (by LMP). It was uncomplicated, and she was asymptomatic till morning, when she woke up in a pool of blood followed by a lower abdominal menstrual-like pain. She denied any history of fever, any systemic illness, any foul smelling vaginal discharge, or any use of medications except prenatal vitamins. Her past medical and surgical history and her family history was not significant, and she denied smoking, use of alcohol or substance abuse.

Physical examination showed that she was alert, conscious and cooperative, pale, and hypotensive (BP: 84/60 mm of Hg) with tachycardia (pulse: 112/min) and temperature 98.4 F. Her systemic examination was unremarkable except the tachycardia and tenderness in the suprapubic area. She has no evidence of any respiratory distress or calf tenderness. Per vaginal examination demonstrated enlarged uterus (16-week size) and some amount of tenderness in the fornix.

Resuscitation with intravenous fluids was started immediately at the emergency department, and blood was sent for cross matching and lab works. Packed red blood cells (RBCs) was started as soon as it was available. Transvaginal sonogram done in the emergency department showed enlarged uterus with intrauterine retained products of conception and also a collection in the pouch of Douglas. Her hemoglobin was 5.5 gm/dl, WBC count 9000/mm^3^, and platelet count 17000/mm^3^. P-time was 100 seconds, aPTT−104 seconds, Fibrinogen <50, D−dimer−980 ng/ml, and her B-HCG in blood was raised. Chest X-ray, urine analysis, and her basic metabolic panel were within normal limits.

After 2 units of packed RBCs, platelet transfusion was started followed by FFP. She was then taken to the operation room, and under aseptic conditions and general anesthesia, suction and evacuation of the retained products followed by dilatation and curettage were done. Hemostasis was checked and patient revived. She was further transfused packed RBCs, platelets, and FFP in the ICU.

Post operative period was uneventful with no evidence of any further bleeding from any sites. She was monitored for any evidence of bleeding and also her hemoglobin and coagulation profile rechecked. In total, she was transfused with 5 units of packed RBCs, 3 units of volume-reduced platelet concentrates, and 4 units of FFP. She had an uncomplicated recovery period and was discharged after 3 days with depot progesterone contraception. The histopathological report of the curettage material later showed products of conception.

## 3. Discussion

Postabortion complications usually are a result of 3 major mechanisms:

- incomplete evacuation of the uterus and uterine atony, which leads to hemorrhagic complications,
- infection,
- Injury due to instruments used during the procedure.

DIC should be suspected in all patients who present with severe postabortion bleeding, especially after mid trimester abortions. Incidence is approximately 200 cases per 100,000 abortions; this rate is even higher for saline instillation techniques (660 per 100,000 abortions) [1].

Disseminated intravascular coagulation (DIC) is an acquired syndrome characterized by the intravascular activation of coagulation with loss of localization arising from different causes. It can originate from and cause damage to the microvasculature, which, if sufficiently severe, can produce organ dysfunction [2]. The resultant clinical condition is characterized by intravascular coagulation and hemorrhage. The affected person is often acutely ill and shocked with widespread hemorrhage (common bleeding sites are mouth, nose, and venepuncture sites), extensive bruising, renal failure, and gangrene. DIC usually presents as an acute, often catastrophic, acquired hemorrhagic tendency. Rarely it can also manifest as a low-grade disorder with predominantly thrombotic manifestations [3]. 

The most common cause of abortion-related DIC is amniotic fluid embolism, which is when amniotic fluid gets into the mother's blood stream. This can be caused by lacerations of the uterus or by compromised blood vessels when the placenta detaches. Infection, either localized in the uterus or generalized (septicemia), can also trigger DIC. Retained products of conception which are most likely to cause DIC when they gets into the maternal bloodstream are fetal brain. There is much debate about the best method of dealing with the fetal head. Some believe that it is best to suction the fetal brain out of the head before crushing it. This eliminates fetal brain matter from the area when the sharp pieces of fetal skull are being removed. These sharp bony fragments are the fetal parts most likely to scratch the cervix and allow fetal tissue to get into the mother's bloodstream.

Others argue that it is sometimes difficult to be sure that the structure you are grasping really is the fetal head and not some part of the mother's body. They recommend squeezing the structure and watching the mother's cervix to see if grey material (fetal brain) oozes out (calvarium show). The sight of “calvarium show" means that the grasped structure is indeed the head of the fetus and it is safe to extract it.

Since DIC can quickly become fatal, it is important that medical professionals recognize the symptoms and diagnose it quickly. Definitive diagnosis depends on the result of [4]

- thrombocytopenia,
- Prolongation of prothrombin time and activated partial thromboplastin time,
- a low fibrinogen concentration,
- increased levels of fibrin degradation products.

The only effective treatment is the reversal of the underlying cause. Anticoagulants are rarely used. Platelets may be transfused if counts are less than 5,000–10,000/mm^3^ and massive hemorrhage is occurring, and fresh frozen plasma may be administered in an attempt to replenish coagulation factors and antithrombotic factors.
